# Gastric emptying assessment in critically ill patients with feed intolerance; comparison of ^13^C octanoic acid, paracetamol and 3-O-methylglucose absorption tests

**DOI:** 10.1186/cc10769

**Published:** 2012-03-20

**Authors:** M Chapman, R Fraser, N Nguyen, A Deane, LS Vasist, K Hacquoil, M Barton, GE Dukes

**Affiliations:** 1 University of Adelaide, Australia; 2 GlaxoSmithKline, Research Triangle Park, NC, USA

## Introduction

Delayed gastric emptying (GE) occurs frequently in critically ill patients and may result in impaired small intestinal delivery of drugs and nutrients. Use of direct methods of GE assessment (scintigraphy) in the ICU for clinical monitoring or research is challeng-ing. Indirect methods that utilize substances which rely on effective GE and rapid absorption from the small intestine offer a feasible estimate of GE. Three substances with these characteristics are ^13^C-octanoic acid (^13^C), paracetamol (PA) and 3-O-methylglucose (OMG). We have previously shown significant correlation to scintigraphy for ^13^C (*r *= 0.63) and OMG absorption (*r *= -0.77 to -0.87). The current study examined the relationship between three indirect methods of GE assessment: ^13^C, PA, OMG.

## Methods

GE was concurrently assessed in mechanically ventilated patients (*n *= 33) with enteral feeding intolerance (gastric residual volume >200 ml) on two occasions 24 hours apart. A test meal of 100 ml Ensure with 100 μl ^13^C, 1,000 mg PA, 3 g OMG was infused into the stomach over 5 minutes. Breath samples for ^13^C and plasma samples for PA, OMG determination were collected over the subsequent 4-hour period. Bivariate Pearson correlations were calculated between the following parameters; ^13^C (gastric emptying coefficient (GEC)), PA (concentration at 60 minutes (C_60_), AUC_0-60 min_), OMG ((C_60_), AUC_0-60 min_). See Table 1.

**Table 1:** Correlations between GE parameters

^13^C GEC	OMG AUC_60_	0.675^*^
^13^C GEC	OMG C_60_	0.758^*^
^13^C GEC	PA AUC_60_	0.678^*^
^13^C GEC	PA C_60_	0.590^*^
PA AUC_60_	OMG AUC_60_	0.534^*^
PA C_60_	OMG C_60_	0.553^*^

*P < 0.0001.

## Results

Observed GE rates for all parameters were across the spectrum from fast to delayed (for example, with *r *range = 0.534 to 0.758, *P *< 0.0001).

## Conclusion

These three practical indirect methods of GE assessment may provide similar estimates of GE in critically ill patients.

